# The taxonomic status of *Petropedetes newtonii* (Amphibia, Anura, Petropedetidae)

**DOI:** 10.3897/zookeys.765.24764

**Published:** 2018-06-06

**Authors:** Alberto Sánchez-Vialas, Marta Calvo-Revuelta, Santiago Castroviejo-Fisher, Ignacio De la Riva

**Affiliations:** 1 Museo Nacional de Ciencias Naturales (MNCN-CSIC), c/ José Gutiérrez Abascal 2, 28006, Madrid, Spain; 2 PUCRS, Pontifícia Universidade Católica do Rio Grande do Sul, Av. Ipiranga 6681, 90619-900, Porto Alegre, Brazil

**Keywords:** Bioko, Cameroon, Equatorial Guinea, morphology, neotype, *Petropedetes johnstoni*, *Petropedetes vulpiae*, *Petropedetes newtonii*, taxonomy

## Abstract

The taxon *Petropedetes newtonii* was described in 1895 by Bocage, from Bioko Island (Equatorial Guinea). This taxon, whose holotype is lost, has been misidentified since Boulenger’s revision of the genus in 1900 and its relationships with other taxa (*P. vulpiae* and *P. johnstoni*) is confusing. Currently, *P. newtonii* is considered a synonym of *P. johnstoni*. In this work, by revising morphological characters of non-webbed *Petropedetes* of Bioko, we demonstrate the morphological singularity of these specimens with respect to *P. johnstoni* and *P. vulpiae* and their association with the name *Petropedetes newtonii*. Consequently, we provide the subsequent designation of a neotype of *P. newtonii* and revalidate this species from its synonym with *P. johnstoni*.

## Introduction

The family Petropedetidae Noble, 1931 includes three genera allopatrically distributed, *Arthroleptides* Nieden, 1911, from East Africa, the monotypic *Ericabatrachus* Largen, 1991, from Ethiopia, and *Petropedetes* Reichenow, 1874, from Central Africa (Frost 2018).

*Petropedetes* is distributed throughout the Gulf of Guinea, in Western Central Africa, including the island of Bioko, where the species generally inhabit the surroundings of fast-flowing streams. Reproduction takes place in land and male parental care has been described (Barej et al. 2010). Tadpoles generally present a semi-terrestrial life stage, developing in the water film covering the surface of rocks or in the water of running streams (De la Riva 1994, Barej et al. 2010). Eight species are currently recognised within the genus, namely: *P. cameronensis* Reichenow, 1874, *P. euskircheni* Barej, Rödel, Gonwouo, Pauwels, Böhme & Schmitz, 2010, *P. johnstoni* (Boulenger, 1888), *P. juliawurstnerae* Barej, Rödel, Gonwouo, Pauwels, Böhme & Schmitz, 2010, *P. palmipes* Boulenger, 1905, *P. parkeri* Amiet, 1983, *P. perreti* Amiet, 1973 and *P. vulpiae* Barej, Rödel, Gonwouo, Pauwels, Böhme & Schmitz, 2010. They are generally easy to distinguish from each other based on the development of webbing and tympanum, and in some dimorphic characters of males such as femoral glands, tympanum size, presence and position of tympanic papilla, and skin keratinised spicules (Barej et al. 2010).

The phylogenetic relationships of the genus *Petropedetes* have been recently revised by Barej et al. (2014), revealing that the species diversity of the genus is underestimated and also that the validity of the taxon *P. newtonii* (Bocage, 1895), placed in the synonymy of *P. johnstoni* by Barej et al. (2010), remains uncertain.

Bocage described *P. newtonii* in 1895 as *Tympanoceros newtonii*. Its description was based on an adult male specimen from Bioko (formerly Fernando Poo) (type locality: “L’île de Fernão do Pó dans le golfe de Guiné”) (Bocage 1895a). The holotype of *P. newtonii* is lost (Perret 1976, Barej et al. 2010) but, fortunately, a detailed illustration and an additional description were published based on a second male specimen collected from Basilé, Bioko (Bocage 1895b). Previous to the description by Bocage (1895a), two more species of *Petropedetes* had already been described: *P. cameronensis* Reichenow, 1874 (type species of the genus) and *P. johnstoni* (= *Cornufer johnstoni* Boulenger, 1888). Boulenger (1900) transferred *Tympanoceros newtonii* (misspelled “*newtoni*”) and *Cornufer johnstoni* to the genus *Petropedetes* and provided the first revision of the genus, with a synthesis of diagnosable characters for the species recognised at that time (*P. cameronensis*, *P. johnstoni*, and *P. newtonii*). The specimens of “*P. newtonii*” used by Boulenger (1900) were from mainland Cameroon and no specimens were included from Bioko (type locality of *P. newtonii*). The morphological characters considered in Boulenger´s revision to characterise *P. newtonii* were not consistent with the holotype description made by Bocage (1895a). Furthermore, Amiet (1983), following Boulenger´s description, studied more characters than those present in the original description of *P. newtonii* (e.g., relative position of tympanic papilla and femoral gland size). Boulenger’s (1900) and Amiet’s (1983) descriptions of *P. newtonii* were widely used by subsequent authors (Mertens 1968, De la Riva 1994, Lasso et al. 2002, Frétey et al. 2012). Incongruences between the original description of *P. newtonii* and the ones using mainland populations (Boulenger 1900, Amiet 1983) have been discussed by Barej et al. (2010). According to Barej et al. (2010), the continental populations of formerly considered *P. newtonii* represent a different evolutionary unit, which was described as *P. vulpiae*. In their revision, Barej et al. (2010) placed *Petropedetes newtonii* (Bocage, 1895) in the synonymy of *P. johnstoni* (Boulenger, 1888), due to the apparent absence of morphological differences between both descriptions. Consequently, two species were considered to occur in Bioko, one of them with webbed toes (*P. cameronensis*) and the other one with non-webbed toes (*P. johnstoni*) (Barej et al. 2010). Specimens of non-webbed *Petropedetes* from Bioko studied using molecular data by Barej et al. (2014) were nested with continental *P. vulpiae*, but forming a clade, together with a few samples from nearby localities in Cameroon, sister to all other samples of *P. vulpiae* (including topotypic specimens). Barej et al. (2010, 2014) indicated that the original description of *P. newtonii* (Bocage, 1895a) does not fit with the morphological characters of *P. vulpiae* and, consequently, they kept them as separate taxa (*P. newtonii* in the synonymy of *P. johnstoni*).

The results of these studies suggest that three independent evolutionary units of *Petropedetes* might be present in Bioko. One of them, *P. cameronensis*, is diagnosable based on molecular and morphological characters and easily recognisable by having males with half-webbed toes, a very small tympanum without tympanic papilla, and metacarpal spine absent. The other two units are non-webbed and correspond to (1) the specimens morphologically assignable to *P. johnstoni* (apparently not studied with molecular data), and (2) the specimens treated as *P. vulpiae* based on molecular data (Barej et al. 2014). The problem posed by Biokoan *P. johnstoni* and *P. vulpiae* in relation to *P. newtonii* needs to be clarified.

The objective of this work is to solve the systematics of the non-webbed *Petropedetes* of Bioko by analysing the morphological characters of the available series of specimens from Bioko included in the molecular phylogeny of Barej et al. (2014) assigned by them to *P. vulpiae*. To do this, we compared the morphological characters of these specimens with (A) the original Bocage’s (1895a) description of *P. newtonii*, Boulenger’s (1888) of *P. johnstoni*, and Barej’s et al. (2010) of *P. vulpiae*; and (B) continental specimens of *P. johnstoni* included in Barej et al.’s (2014) molecular study. As a result, we demonstrate the morphological singularity of the non-webbed *Petropedetes* from Bioko with respect to both *P. johnstoni* and *P. vulpiae* and the association of these *Petropedetes* from Bioko with the original *P. newtonii*. Consequently, we revalidate the taxon *P. newtonii* from its junior synonym with *P. johnstoni* and designate and describe a neotype of *P. newtonii* to allow its fully taxonomic recognition and facilitate future work on *Petropedetes*.

## Materials and methods

We revised 14 specimens of non-webbed *Petropedetes* from several localities of Bioko (Equatorial Guinea) held at the herpetological collection of the Museo Nacional de Ciencias Naturales (MNCN-CSIC), Madrid, Spain. Three specimens (MNCN 46703, MNCN 46708, and MNCN 46719) were collected in March 2007 and preserved in 70 % ethanol. The other 11 specimens were collected in November and December 2003, fixed in formalin 10 % and preserved in ethanol 70 %. Prior to fixation, a piece of tissue was preserved in ethanol 96 % and stored in a freezer for molecular studies. Among them, three specimens were previously included in the molecular analysis of Barej et al. (2014): MNCN 48728 (MNCN-DNA 50405), MNCN 48729 (MNCN-DNA 50465) and MNCN 48730 (MNCN-DNA 50411). Additional specimens of *Petropedetes johnstoni* stored in 70 % ethanol and housed at the Zoologisches Forschungsmuseum Alexander Koenig (ZFMK 87709, adult male, and ZFMK 87710, adult female) were studied. These specimens were collected from Nkoelon, Campo region (2°23'49.8"N, 10°02'47.4"E), Cameroon. Studied specimens and their associated data are listed in Table 1. Nomenclature used in the morphological description of the neotype follows De la Riva et al. (2012).

**Table 1.** Examined specimens of *Petropedetes*. Morphometric measurements are given in mm. Abbreviations: SVL (snout-vent length), HL (head length, from rictus to point of snout), HW (head width, at level of rictus), IND (internarial distance), END (distance from eye to nostril), HTD (horizontal tympanum diameter), ED (eye diameter), NS (distance from nostril to snout tip), FL (femur length), FGL (femoral gland length), FGW (femoral gland width), TL (tibia length), FTL (foot length, from proximal border of inner metatarsal tubercle to tip of fourth toe), THL (thenar tubercle length), and THBL (thumb length).

Species	Voucher number	Field Code	Country	Main political unit	Locality	Latitude	Longitude	Elevation (m)	Sex	SVL	HW	HL
*P. newtonii*	MNCN 46703	RC.3.1	Equatorial Guinea	Bioko Island	Campamento Smith, Río Tudela	3°18'27.34"N, 8°28'15.68"E		181	FEMALE	40.4	16.8	16.3
*P. newtonii*	MNCN 46708	RC.10	Equatorial Guinea	Bioko Island	BBPP Camp, Caldera de Luba	3°20'45.34"N, 8°29'48.03"E		871	INDET/JUV	28.9	12.1	12.6
*P. newtonii*	MNCN 48728	ET105	Equatorial Guinea	Bioko Island	Chopepe creek on its confluence with Río Osa	3°14'52.19"N, 8°32'23.77"E		27	MALE	34.9	15.5	14.1
*P. newtonii*	MNCN 48730	ET113	Equatorial Guinea	Bioko Island	Afluent of Río Olé on right margin near camp Bite on tarck to Caldera de Luba	3°18'27.08"N, 8°28'24.36"E		254	MALE	32.5	14.4	14.5
*P. newtonii*	MNCN 48729	ET579	Equatorial Guinea	Bioko Island	Río Sibitá, Bococo Avendaño	3°26'46.04"N, 8°26'52.39"E		33	FEMALE	41.3	16.6	15.5
*P. newtonii*	MNCN 48955	ET112	Equatorial Guinea	Bioko Island	Afluent of Río Olé on right margin near camp Bite on tarck to Caldera de Luba	3°18'27.08"N, 8°28'24.36"E		257	FEMALE	41.3	16.2	15.5
*P. newtonii*	MNCN 48957	ET580	Equatorial Guinea	Bioko Island	Río Sibitá, Bococo Avendaño	3°26'46.04"N, 8°26'52.39"E		33	INDET/JUV	30.3	12.4	11.6
*P. newtonii*	MNCN 48956	ET107	Equatorial Guinea	Bioko Island	Chopepe creek on its confluence with Río Osa	3°14'52.19"N, 8°32'23.77"E		27	FEMALE	29.7	11.3	11.6
*P. newtonii*	MNCN 48960	ET 119	Equatorial Guinea	Bioko Island	BBPP Camp, Caldera de Luba	3°20'47.32"N, 8°29'48.44"E		875	INDET/JUV	16.3	6.6	7.3
*P. newtonii*	MNCN 48961	ET 108	Equatorial Guinea	Bioko Island	Chopepe creek on its confluence with Río Osa	3°14'52.19"N, 8°32'23.77"E		27	INDET/JUV	14.6	6.0	6.1
*P. newtonii*	MNCN 48962	ET 84	Equatorial Guinea	Bioko Island	Stream Mukokobe. Path between Belebu and Ureka	3°24'25.81"N, 8°33'3.23"E		895	INDET/JUV	10.1	4.6	4.6
*P. newtonii*	MNCN 46719	RC.19	Equatorial Guinea	Bioko Island	Río Riaco	3°20'31.83"N, 8°29'59.01"E		834	INDET/JUV	14.4	5.5	5.4
*P. newtonii*	MNCN 48958	ET 103	Equatorial Guinea	Bioko Island	Chopepe creek on its confluence with Río Osa	3°14'52.19"N, 8°32'23.77"E		27	INDET/JUV	17.5	7.1	7.0
*P. newtonii*	MNCN 48959	ET106	Equatorial Guinea	Bioko Island	Chopepe creek on its confluence with Río Osa	3°14'52.19"N, 8°32'23.77"E		27	INDET/JUV	16.8	7.3	7.9
*P. johnstoni*	ZFMK87709	N/A	Cameroon	South Region	Nkoelon, Campo region	2°23'49.8"N, 10°02'47.4"E		115	MALE	32.4	14.0	12.5
*P. johnstoni*	ZFMK87710	N/A	Cameroon	South Region	Nkoelon, Campo region	2°23'49.8"N, 10°02'47.4"E		115	FEMALE	38.0	14.6	13.8

**Table 1.** Continued.

Species	Voucher number	IND	FL	FGL	FGW	TL	FTL	HTD	ED	END	NS	THL	THBL	Microhabitat	Date	Time
*P. newtonii*	MNCN 46703	3.6	22.1	6.7	2.6	22.4	31.1	2.8	6.4	4.3	2.8	2.0	6.3	N/A	3/7/2007	N/A
*P. newtonii*	MNCN 46708	3.2	16.1	5.0	2.0	17.5	23.6	2.1	4.1	3.4	1.8	1.5	6.5	N/A	3/10/2007	N/A
*P. newtonii*	MNCN 48728	3.7	20.8	6.9	4.8	21.3	25.9	3.2	5.7	4.6	2.4	2.2	5.7	On a leaf (20 × 15 cm) 35 cm above the ground and 1 m from water	11/22/2003	19:00
*P. newtonii*	MNCN 48730	3.0	18.6	7.1	3.9	18.5	24.5	2.5	5.4	4.0	2.1	2.1	4.2	On the shore on a rock	11/25/2003	19:30
*P. newtonii*	MNCN 48729	4.0	20.3	4.7	2.3	21.8	28.9	3.1	6.4	4.0	2.5	2.1	6.6	On a leaf (40 × 10 cm) 30 cm above water of 20 cm deepth	12/3/2003	18:45
*P. newtonii*	MNCN 48955	3.7	21.7	6.9	2.8	22.0	27.7	2.6	6.8	3.9	2.1	2.4	6.5	On a rock (60 × 80 cm) in the middle of the water 20 cm above near MNCN 48730	11/25/2003	19:25
*P. newtonii*	MNCN 48957	3.2	18.1	3.9	2.0	18.9	N/A	2.0	4.6	3.5	2.1	1.8	5.2	On a dry leaf (20 × 15 cm) 7 cm above Rio Sibitá of 5 cm deepth	12/3/2003	18:45
*P. newtonii*	MNCN 48956	3.0	15.9	4.1	1.8	16.3	21.6	1.9	4.2	3.5	2.0	1.7	4.9	On a branch (1 cm diameter) about 1.80 m above water	11/22/2003	19:00
*P. newtonii*	MNCN 48960	2.3	8.0	N/A	N/A	8.5	7.9	1.0	3.0	1.9	1.0	N/A	2.0	On the ground of the kitchen, no vegetation and no body of water in the surroundings	11/26/2003	12:30
*P. newtonii*	MNCN 48961	2.0	7.8	N/A	N/A	8.9	6.4	0.8	2.7	1.8	0.9	N/A	2.0	On top of a leaf (20 × 10 cm), 1 m above the water, of a plant growing on a rock of the stream	11/22/2003	19:30
*P. newtonii*	MNCN 48962	N/A	6.0	N/A	N/A	6.0	5.4	1.1	2.0	N/A	N/A	N/A	N/A	On the moss covering a rock of the stream	20/11/003	9:30
*P. newtonii*	MNCN 46719	1.6	7.8	N/A	N/A	7.6	6.5	0.8	2.7	1.3	0.9	N/A	1.5	N/A	3/15/2007	N/A
*P. newtonii*	MNCN 48958	1.9	9.7	2.0	1.0	10.0	9.2	1.2	2.6	1.9	1.0	N/A	2.6	On the ground, a mix a mud and leaf-litter, 2 m from the water	11/22/2003	17:30
*P. newtonii*	MNCN 48959	2.3	9.5	2.8	1.2	10.6	8.2	1.0	2.8	1.8	1.0	N/A	2.3	Over a small leaf (7 × 4 cm) 10 cm above ground and 0.5 m from the water	11/22/2003	19:00
*P. johnstoni*	ZFMK87709	3.5	18.4	7.9	3.4	20.1	25.0	2.9	5.6	3.2	1.4	1.7	4.6	N/A	N/A	N/A
*P. johnstoni*	ZFMK87710	4.0	20.3	5.6	2.4	21.9	26.2	3.0	5.8	3.6	1.7	1.7	5.3	N/A	N/A	N/A

Measurements were taken with a digital caliper to the nearest 0.1 mm, and are given in mm. Morphometric abbreviations are as follows:

**SVL** (snout-vent length)

**HL** (head length, from rictus to point of snout)

**HW** (head width, at level of rictus)

**IND** (internarial distance)

**END** (distance from eye to nostril)

**TD** (horizontal tympanum diameter)

**ED** (eye diameter)

**NS** (distance from nostril to snout tip)

**FL** (femur length)

**FGL** (femoral gland length)

**FGW** (femoral gland width)

**TL** (tibia length)

**FTL** (foot length, from proximal border of inner metatarsal tubercle to tip of fourth toe)

**THL** (thenar tubercle length)

**THBL** (thumb length)

For qualitative morphological diagnosis, we selected male specimens, which possess the most important characters to differentiate these species. These features are: tympanum size, presence of tympanic papilla and its relative position in the tympanum, presence of keratinised spicules on basis of arms, relative size of femoral glands and webbing development.

Digital photographs were taken with a reflex camera fitted with a macro lens. Micro-computed tomography (micro-CT) scans were carried out for two specimens (male and female) of non-webbed *Petropedetes* from Bioko (MNCN 48728, MNCN 48729) and in the same way for two specimens (male and female) of *P. johnstoni* (ZFMK 87709, ZFMK 87710), at the MNCN. The scans were produced using a XTH 160 Nikon Metrology, with a molybdenum target. Specimens were scanned with the following settings: 126 kV, and 47 µA over 1000 projections during 1.30 h. Raw X-ray data were processed using CTPro 3D software (Nikon Metrology) and micro-CT images were analysed using VG Studio MAX 3.0.3 (Volume Graphics, Heidelberg, Germany).

## Results and discussion

### *Petropedetes johnstoni* and *P. newtonii* original descriptions

*Petropedetes johnstoni* was described by Boulenger in 1888, based on a subadult specimen from Río del Rey, Cameroon (“Cameroons”) (= river Ndian, Western Cameroon). Despite it was treated as a female by Boulenger (1888), Parker’s (1936) revision of the holotype stated that it corresponds to an immature male (Amiet 1983).

Bocage (1895a) described *Petropedetes newtonii* from Bioko based on a single adult male specimen with well-marked sexual features.

We agree with Barej et al. (2010), which stated that morphological discrepancies between both descriptions are due to ontogeny, measurement methods (as for the tympanum size), and intraspecific variability (as for the relative position of the tibiotarsal articulation in regard to the snout tip when the leg is stretched forwards). While the holotype of *P. johnstoni* is a subadult male which lacks the typical male secondary characters, the holotype description of *P. newtonii* resembles that of a male possessing reproductive features. Barej et al. (2010) argue that both descriptions represent morphologically indistinguishable taxa and, with the evidence at hand, considered *P. newtonii* a junior synonym of *P. johnstoni*. However, we argue that this decision needs to be evaluated in the light of detailed comparisons of adult male specimens, which requires, for reasons outlined above, the study of additional specimens besides the type series. These results are presented in the following sections.

### Morphological revision of *P. johnstoni*

Two specimens of *P. johnstoni* (ZFMK 87709, ZFMK 87710) were morphologically revised in order to complete the original description of the holotype made by Boulenger (1888). The selected specimens were previously characterised based on molecular data by Barej et al. (2014) and are unambiguously nested in the clade of *P. johnstoni*, which includes samples from the type locality of *P. johnstoni*.

**Figure 1.**
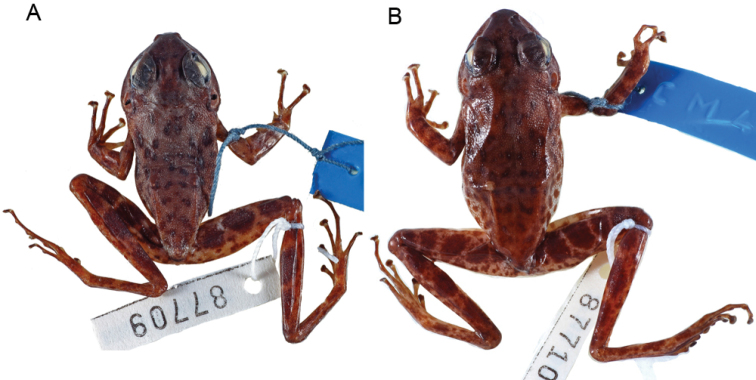
Dorsal view of *Petropedetes johnstoni* specimens (**A** male, ZFMK 87709 **B** female, ZFMK 87710).

**Figure 2.**
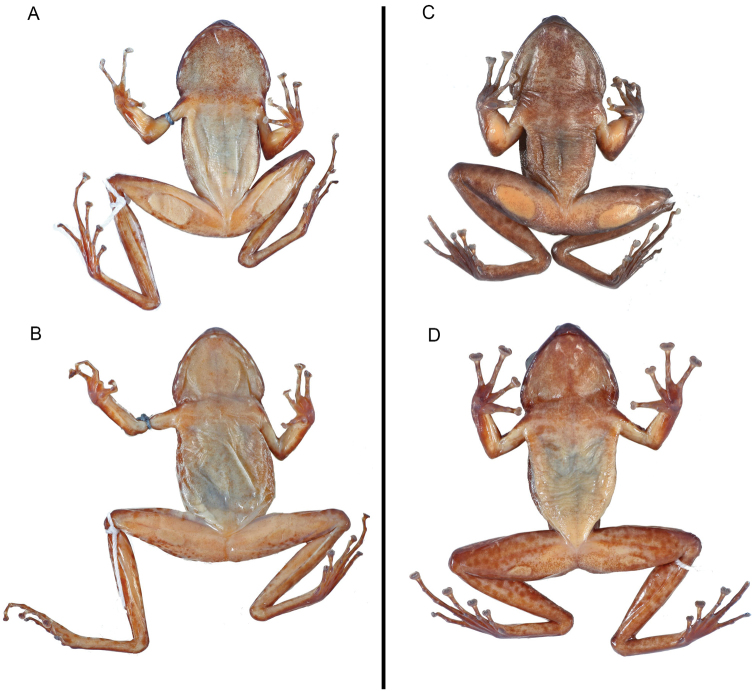
Ventral view of specimens of *Petropedetes johnstoni* (**A** male, ZFMK 87709 **B** female, ZFMK 87710) and *P. newtonii* (**C** male, MNCN 48730 **D** female, MNCN 48729).

**Figure 3.**
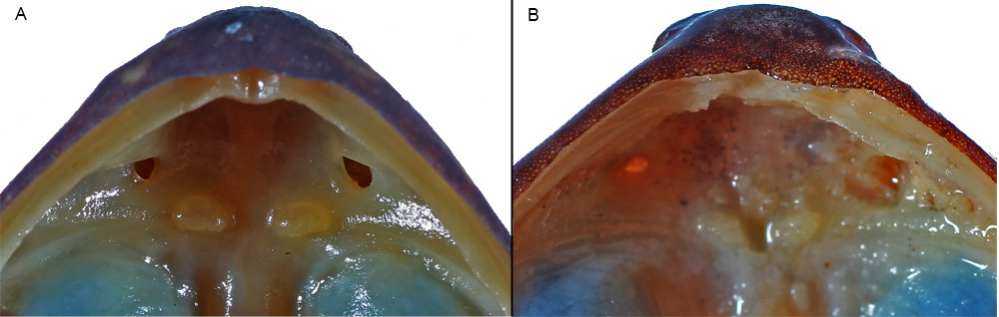
Mouth roof region of *Petropedetes newtonii* (**A** female, MNCN 48729) and *Petropedetes johnstoni* (**B** male, ZFMK 87709).

**Figure 4.**
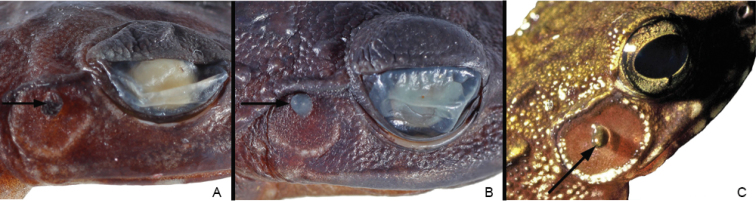
Details of the tympanum of *Petropedetes johnstoni* (**A** male, ZFMK 87709), *P. newtonii* (**B** male, MNCN 48728), and *P. vulpiae* (**C** male, non-collected specimen from Río Muni, Equatorial Guinea; photo by Ignacio De la Riva).

**Figure 5.**
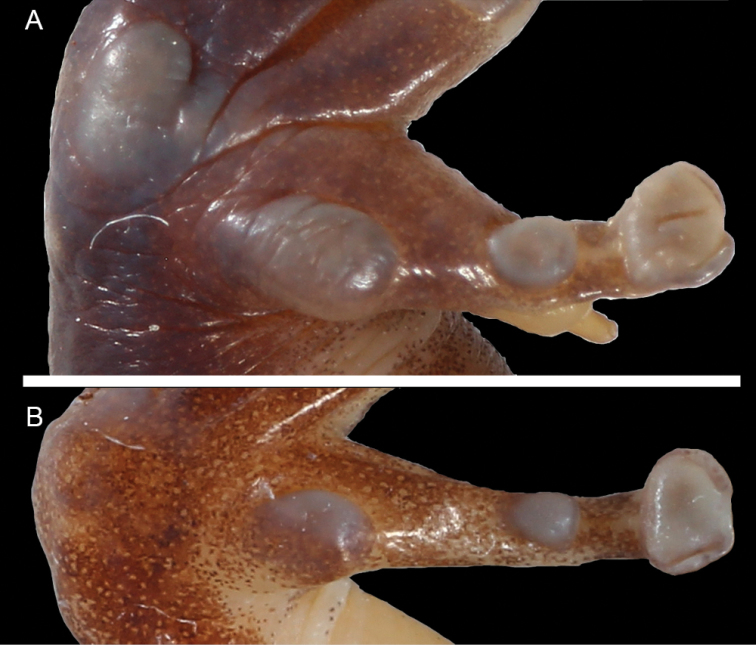
Thenar tubercle of males of *Petropedetes newtonii* (**A** male, MNCN 48728) and *P. johnstoni* (**B** male, ZFMK 87709). Note the different shape and size and the presence of a dorsal spine on the distal edge of the metacarpal of Finger I in *P. newtonii*.

Both specimens are characterised by: whitish posterior side of the thighs with a speckled pattern made up of brownish dots or marks (Fig. 1); ventral side whitish; lower jaw rounded, with several well marked white spots; snout slightly rounded in dorsal and ventral view (Figs 1, 2A, B); relatively small vomerine teeth (Fig. 3B); supratympanic fold distinct (Fig. 4A); palmar tubercle oval (Fig. 5B); thenar tubercles in both specimens oval (Fig. 5B), approximately ¼ of total length of Finger I; one subarticular tubercle on Finger I, placed between fingertip and centre of the finger; subarticular tubercle of Finger II centrally positioned on the finger; fingers III and IV with two subarticular tubercles; fingertips with adhesive discs of different sizes, as follows: I > II > III = IV; relative lengths of fingers: III > IV > II > I; toes not webbed or rudimentarily; outer metatarsal tubercle absent; inner metatarsal tubercle elongated, at the base of Toe I. Toes I and II with one single tubercle, toes III and V with two single tubercles and Toe III with three single tubercles; no supernumerary tubercles; relative lengths of toes: IV > III > V > II > I; skin with relatively small warts, especially on the anterior part of dorsum. Morphometric measurements are given in Table 1.

**Figure 6.**
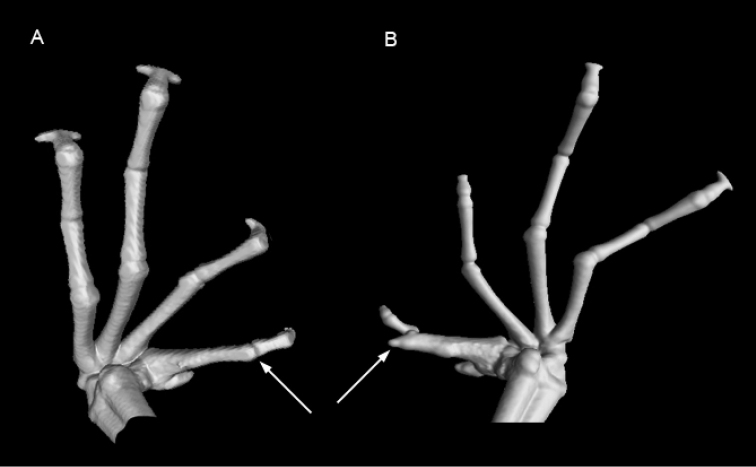
CT-scan image of the dorsal side of the hands of *Petropedetes johnstoni* (A male, ZFMK 87709) and *P. newtonii* (**B** male, MNCN 48728). No traces of metacarpal spines are detected in *P. johnstoni*.

Male specimen ZFMK 87709 is characterised by possessing a small tympanum (relation of the TD to ED = 0.53), tympanic papilla present in the upper border of the tympanum (Fig. 4A), a big femoral gland covering most of the femoral skin (relation of femoral gland to femur length = 0.43; Fig. 2A), lack of keratinised spicules on the skin of the basis of arms (Fig. 2A), upper and lower border of tympanum round, not flattened (Fig. 4A), forearm not hypertrophied (Fig. 2A), metacarpal spines absent (Fig. 6A) and webbing rudimentary.

**Figure 7.**
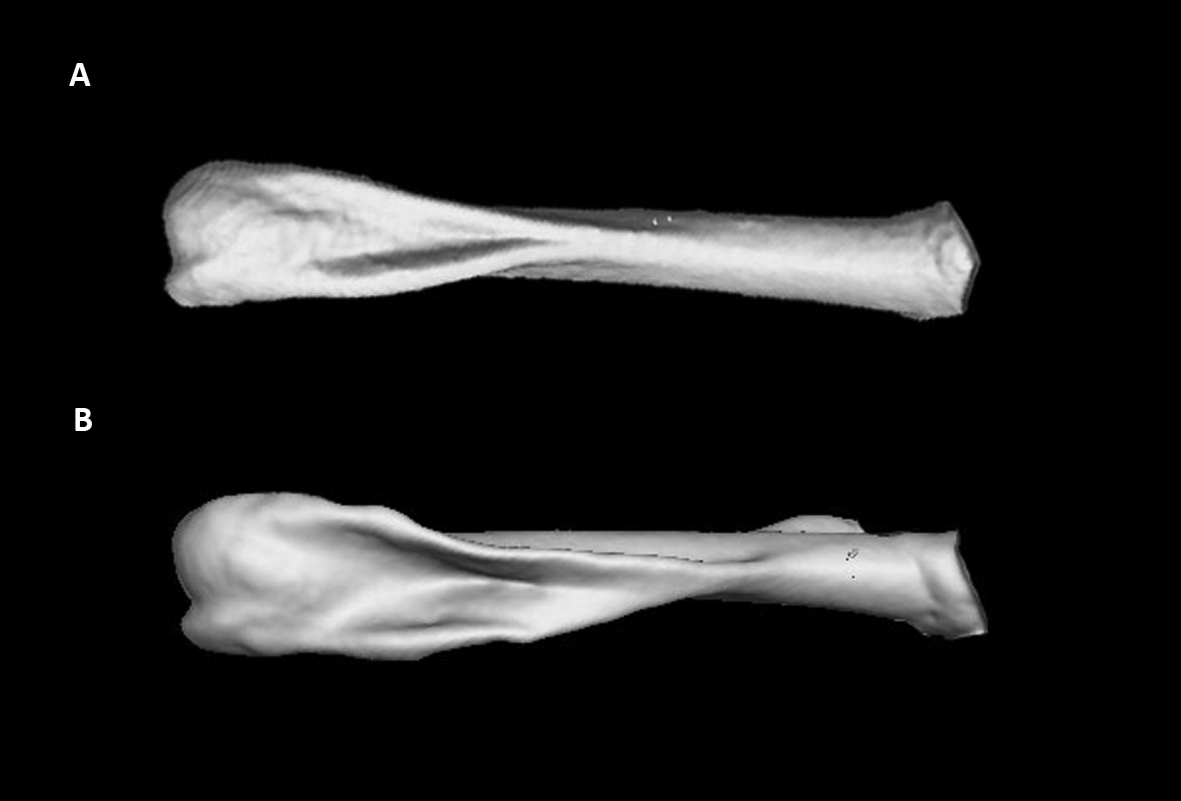
CT-scan image of the dorsal side of the humerus of *Petropedetes johnstoni* (**A** male, ZFMK87709) and *P. newtonii* (**B** male, MNCN 48728). Note the differences in the development and extension of the bone crest.

The study of the humerus by CT-scan analyses of a male (ZFMK 87709; Fig. 7A) and a female (ZFMK 87710) of *P. johnstoni*, shows a strong sexual dimorphism due to the presence in the male of a double crest, relatively short in length and distally divergent in the dorsal side of the bone, which is totally absent in the female.

### Summary of the diagnostic characters of *P. vulpiae*

The other species involved in the taxonomic problem of *Petropedetes newtonii*, and recently suggested to be in Bioko on the basis of DNA sequences (Barej et al. 2014), is *P. vulpiae*. Before we proceed with a description of the morphology of the new material of non-webbed *Petropedetes* from Bioko, we summarised the main characters of this species to allow a clearer discussion. The diagnosis made by Barej et al. (2010) reads as follows: “medium sized *Petropedetes*; compact body shape; tympanum usually flattened on the upper and lower border; tympanum larger than diameter of eye in males, smaller in females; characters of breeding males: tympanic papilla present (broad, fleshy), papilla closer to the centre than the upper border; forearm hypertrophy well developed; carpal spike present; spinosities on throat, forearms and on almost every wart on flanks and dorsum, even around the tympanum; femoral glands large, very prominent; webbing rudimentary”. Also, comparative drawings of some features as femoral glands and tympanum of *P. vulpiae* were included in Barej et al.’s (2010) revision, illustrating the most relevant characters and allowing indirect observations.

### Description of the new material of non-webbed *Petropedetes* from Bioko

**Figure 8.**
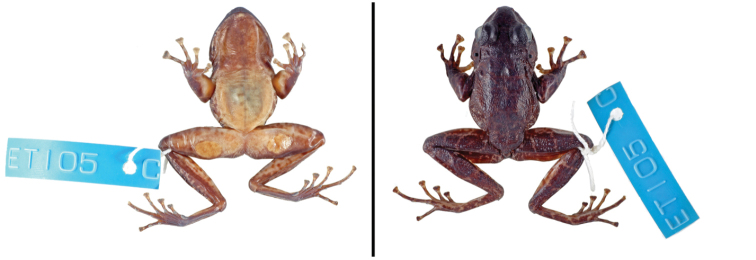
Neotype of *Petropedetes newtonii* (adult male, MNCN 48728). Left: ventral view; right: dorsal view.

**Figure 9.**
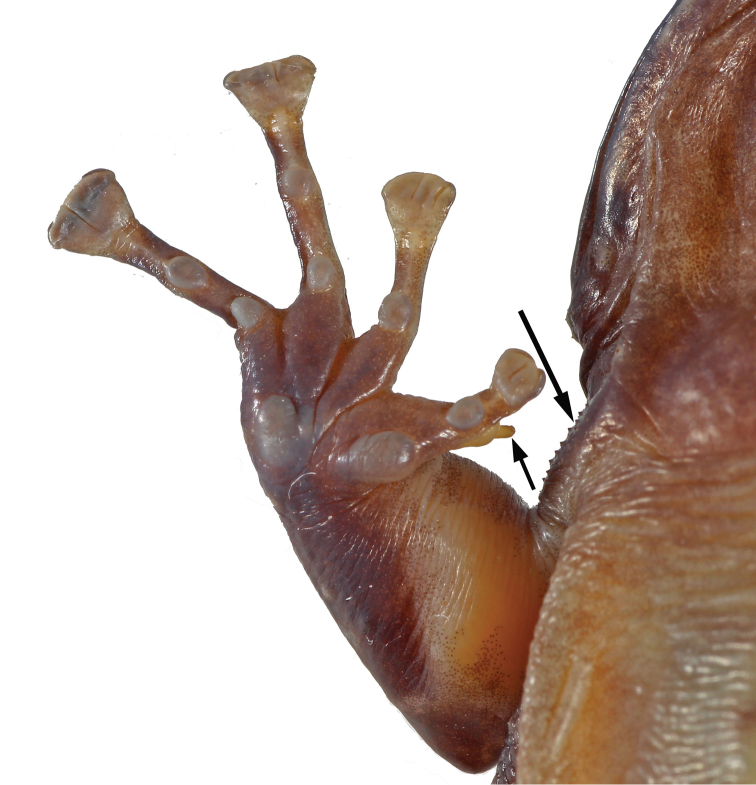
Ventral view of the right forelimb of *Petropedetes newtonii* (adult male, MNCN 48728). Arrows point to the dorsal spine on the distal edge of the metacarpal of Finger I and keratinised spicules along the skin surface of the upper arm and labial commissure.

We revised the morphology of a series of 14 specimens (8 juveniles, 4 adult females, 2 adult males) collected in Bioko, including the three individuals studied in the molecular phylogeny of Barej et al. (2014). The adults have the following characteristics: Lower jaw relatively triangular; snout slightly pointed in dorsal and ventral view (Figs 2C, D, 8); relatively big vomerine teeth (Fig. 3A); supratympanic fold distinct (Fig. 4B); palmar tubercle present, oval (Fig. 9). Thenar tubercle elongated, more than 1/3 of Finger I total length (Figs 5A, 9); one subarticular tubercle on Finger I, placed between fingertip and the centre of the finger; subarticular tubercle of Finger II centrally positioned on the finger; fingers III and IV with two subarticular tubercles, fingertips with adhesive discs of different sizes, as follows: I > II > III = IV; relative lengths of fingers: III > IV > II > I; toes not webbed or rudimentarily; outer metatarsal tubercle absent; inner metatarsal tubercle elongated, on the base of Toe I. Toes I and II with one single tubercle, toes III and V with two single tubercles and Toe III with three single tubercles; no supernumerary tubercles; relative lengths of toes: IV > III > V > II > I. Skin with relatively medium-sized warts, especially on the anterior part of dorsum. Morphometric measurements are given in Table 1, including two grown juveniles.

Both adult male specimens (MNCN 48728 and 48730) are characterised by sharing the following features: (1) the size of the tympanum (relation between TD and ED = 0.56 and 0.46 mm respectively), which is approximately half size of the eye diameter; (2) the position of the tympanic papilla in the upper border of tympanum (Fig. 4B); (3) the tympanum upper and lower borders round, not flattened; (4) the relatively large femoral glands (Figs 2C, 8), covering a big area of the femoral skin (relation of femoral gland to femur length = 0.33 and 0.38, respectively); (5) the presence of dispersed keratinised spicules on the skin of the basis of forelimb, inner surface of upper arm, lower tympanic region and supratympanic fold, and on the postcommissural region (more distinct in MNCN 48728; Fig. 9); (6) the hypertrophied, well developed forelimb (Figs 8, 9); (7) the dorsal spine on the distal edge of the metacarpal of Finger I (Figs 5A, 6B, 9); and (8) the rudimentary webbing (Fig. 9).

The study of the humerus by CT-scan of a male (MNCN 48728; Fig. 7B) and a female (MNCN 48729) shows a strong sexual dimorphism due to the presence in the male of a well-developed double crest, distally divergent in the dorsal side of the bone, which is totally absent in the female.

### Comparison of *P. johnstoni*, *P. vulpiae*, and non-webbed *Petropedetes* from Bioko

Considering the descriptions provided above, the specimens from Bioko could represent: (i) a new species yet to be named under the rules of the ICZN; (ii) part of *P. vulpiae* as suggested by the phylogenetic analyses of DNA sequences by Barej et al. (2014); (iii) part of *P. newtonii* as a synonym of *P. johnstoni*; or (iv) part of *P. newtonii* as a valid species. We argue in favour of the last scenario.

Our description of non-webbed *Petropedetes* from Bioko is fully concordant with the description of *P. newtonii*. In other words, none of the characters described in the original description of *P. newtonii* is incompatible with our own observations. Considering the geographic relationship between the specimens (both from Bioko) and their morphological similarity, we consider these specimens part of *P. newtonii*. Furthermore, our detailed study of external and internal morphology of specimens of both *P. johnstoni* and *P. newtonii* led us to discover a number of important differences (Table 2): (i) absence of keratinised spicules on the skin of the throat and on the basis of the arms in *P. johnstoni* (see also Amiet 1983), which are present in *P. newtonii*; (ii) absence of metacarpal spines in the adult male of the revisited specimen of *P. johnstoni* (Fig. 6), which are present in studied specimens of *P. newtonii*; (iii) specimens of both sexes of *P. johnstoni* present a lower jaw and a snout more rounded than specimens of *P. newtonii*; (iv) thenar tubercles are oval and distinctly smaller in *P. johnstoni* than in studied specimens of *P. newtonii*; and (v) the vomerine teeth are smaller in *P. johnstoni* than in studied specimens of *P. newtonii*, and transversally oriented (Fig. 3). Based on these differences, we consider *Petropedetes newtonii* a valid taxon and revalidate it from its junior synonym with *P. johnstoni*.

**Table 2.** Morphological data and character states for the studied *Petropedetes* species.

Species	*P. vulpiae*	*P. johnstoni*	*P. newtonii*
**Male tympanum size**	Bigger than eye diameter	Smaller than eye diameter	Smaller than eye diameter
**Tympanic papilla position**	Close to the centre	Close to upper border	Close to upper border
**Tympanum upper border shape**	Flattened	Rounded	Rounded
**Dorsal metacarpal spine**	Present	Absent	Present
**Skin keratinised spicules**	Present	Absent	Present
**Male humerous crest**	Unknown	Relatively short	Long and well developed
**Snout shape**	Slightly pointed	Slightly rounded	Slightly pointed
**Thenar tubercle lenght**	Unknown	Shorter than 1/3 of the finger I	Longer than 1/3 of finger I

*Petropedetes vulpiae* is easily distinguishable from *P. johnstoni* and *P. newtonii*, sensu this work, based on the sexual dimorphic characters present in reproductive males (Table 2). *Petropedetes vulpiae* possesses a bigger tympanum, flattened on its upper and lower borders (Fig. 4), and the papilla is closer to the centre of the tympanum. The results of the phylogenetic analyses Barej et al. (2014) are puzzling. The Biokoan specimens studied by us and herein assigned to *P. newtonii* that are included in Barej et al. (2014) cluster with three purported specimens of *P. vulpiae* from nearby continental Cameroon, with nearly identical haplotypes. However, topotypic samples of *P. vulpiae* are located in the sister clade of the Biokoan specimens together with samples from other localities of Cameroon and Nigeria (Barej et al. 2014). To solve the incongruence between the morphological and molecular data at hand, we reidentified the specimens of *P. vulpiae* ZMB 78421, NMP6V 73439/1 and 73439/1 sensu Barej et al. (2014) as *P. newtonii*. Thus, *P. newtonii* and *P. vulpiae* are reciprocally monophyletic sister species.

### Designation and description of the neotype of *Petropedetes newtonii*

The morphological and molecular distinctiveness of *Petropedetes* from Bioko in relation to *P. vulpiae* and *P. johnstoni* are clear. As the type material of *P. newtonii* is lost, we deem it necessary to designate a neotype.

*Tympanoceros newtonii* Bocage, 1895: 270, bona species. Terra typica: “L’île de Fernão do Pó dans le golfe de Guiné”.

*Petropedetes newtoni* –Boulenger 1900: 439 (misspelled).

**Neotype.** An adult male in the collection of the Museo Nacional de Ciencias Naturales (Madrid, Spain), MNCN 48728, field number ET105, collected on 22 November 2003 by Santiago Castroviejo-Fisher at Chopepe creek at its confluence with Río Osa (3°14'52.19"N, 8°32'23.77"E, 27 m a.s.l.), Bioko, Equatorial Guinea (Fig. 9).

**Description.** Measurements (in mm) are listed in Table 1. Adult male specimen of medium size (SVL 34.9 mm) in good state of preservation, with distant phalange of Toe IV cut for molecular analyses; body relatively robust; head slightly wider than long (head width 44.4 % of SVL; head length 40.4 % of SVL). Head moderately triangular in shape; snout relatively pointed in dorsal and ventral view; nostrils protuberant, laterally oriented and very close to tip of snout; internarial distance 3.7 mm (23.9 % of HW); nostrils closer to tip of snout than to margin of orbit; internarial region slightly concave; eye very large, 40.3 % of head length; interorbital distance 2.4 mm; loreal region highly concave; tympanum distinct, rounded, not flattened in its upper side; tympanic papilla present, located in the upper margin of tympanum; supratympanic fold distinct, extending from behind the eye, bordering the tympanum, to close the level of shoulder; choanae moderately large, subcircular; vomerine teeth present, relatively big and close to each other, transversally positioned between choanae and eye orbit; tongue elongated, oval, cordiform (heart-shaped, notched posteriorly) and with a single papilla located in the middle of the anterior region.

Forelimb robust; forearm hypertrophied; paired humeral crest high, extending over most of humerus length; relative lengths of fingers III > IV > II > I; palmar webbing absent; tips of fingers flat, expanded as adhesive discs; adhesive discs heart-shaped, with two oval plates on the dorsal side; relative width of terminal discs IV = III > II > I; terminal phalanges T-shaped; thenar (inner palmar) tubercle oval, more than 1/3 of Finger I total length; outer palmar tubercle distinct, rounded, bigger than thenar tubercle; one subarticular tubercle on Finger I, placed between fingertip and the centre of the finger; subarticular tubercle of Finger II centrally positioned; fingers III and IV with two subarticular tubercles; dorsal spine on the distal edge of the metacarpal of Finger I robust, whitish; keratinised spinules on humeral skin present. Hind limbs moderately robust and long (femur and tibia length 42.1 mm); femoral gland large, subcircular, 33.5 % of femur length; toes not webbed or rudimentarily; tips of toes flat, expanded as adhesive discs; adhesive discs heart-shaped, with two oval plates on the dorsal side; relative width of terminal discs: II > I > III > IV > V; terminal phalanges T-shaped; toes long; relative length of toes: IV > III > V > II > I; toes I and II with one single tubercle, toes III and V with two single tubercles and Toe III with three single tubercles; no supernumerary tubercles; outer metatarsal tubercle absent; a distinct, elongated inner metatarsal tubercle.

Skin of dorsum with scattered pustules, especially distinct in the anterior region at the level of the shoulder; keratinised spicules on inner surface of upper arm, lower tympanic region, supratympanic fold, and on postcommissural region; ventral skin smooth.

Coloration of dorsal surfaces in preservative dominated by different brown tonalities, with whitish transversal lines or marks dispersed on hind and fore limbs; posterior margin of finger and toe tips whitish. Ventral coloration white, except in the throat, palmar surfaces, and tibia, which are brownish; inner side of forearms white, external side brown; ventral side of hind legs whitish with dispersed, brown rounded spots.

### Distribution of *Petropedetes newtonii*

There are few geographical records published of *P. newtonii* *sensu stricto.* The original type locality lacks a specific location in Bioko; however, the second known specimen was collected in Basilé, Bioko Norte province (“Bassilé”, at 527 m asl. on the grass [Bocage 1895b]). Posteriorly, other populations of *P. newtonii* were recorded within Bioko Sur province: Musola (Boulenger 1906), Rio Iladyi, at 1000 m asl. (Mertens 1965), and another in Bioko Norte province without specific locality: NW Bioko (Bocage 1903). Barej et al. (2014) provided the following specific localities: Río Osa, Río Ole, Río Sibitá and Caldera de Luba, based on specimens held at the Museo Nacional de Ciencias Naturales, all collected by SC-F in 2003 (Table 1). Additonally, Barej et al. (2014) studied populations from the southern coast of Cameroon, which are nested within the clade of Biokoan populations of *P. newtonii* (considered as *P. vulpiae* by Barej et al. [2014]).

**Figure 10.**
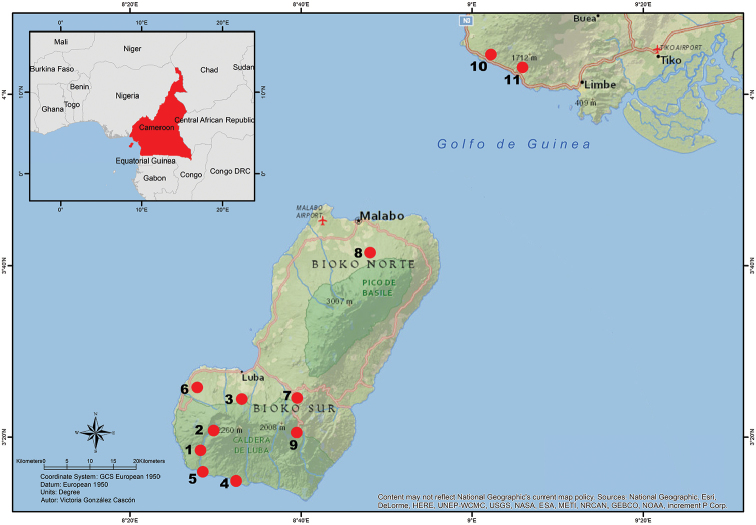
Distribution map of *Petropedetes newtonii* based on published records and collection data. **1** Campamento Smith, Luba, Bioko **2** Río Riaco, Caldera de Luba, Bioko **3** Stream Mukokobe, between Belebu and Ureka, Bioko **4** Río Osa, Bioko **5** Río Ole, Bioko **6** Río Sibitá, Bococo, Bioko **7** Musola, Bioko **8** Basilé, Bioko **9** Río Iladyi, Bioko **10** Bakingili, Cameroon **11** Mt. Etinde, Cameroon.

Therefore, populations from continental Africa like those from Río Muni (Equatorial Guinea), that were formerly considered as *P. newtonii* (De la Riva 1994, Lasso et al. 2002) are now included in the taxon *P. vulpiae* (Barej et al. 2010). The distribution of *P. newtonii* would be formed by widely distributed populations throughout Bioko (both in Bioko Norte and Bioko Sur) and by continental populations on the southern coast of Cameroon (Fig. 10).

As a consequence of this validation, it must be said that the tadpole of *P. newtonii* has been described by Barej et al. (2010) (as *P. johnstoni*) based on a specimen from Musola, Bioko, which was cited by Boulenger (1906).

**Natural history.** Descriptions are based on the field notes of SC-F (Table 1). All specimens were collected after dusk between 18:45 and 19:30, the furthest distance from the water 2 m, in the shallower parts (20 cm depth) of streams between 22 November and 03 December 2003, which typically corresponds to the rainy season. The exceptions are three juveniles (MNCN 48960, 48962, and 48958) found during the day, one of them (MNCN 48960) in a place with no body of water in the immediate surroundings (Table 1). Two adult specimens (male MNCN 48730 and gravid female MNCN 48955) collected on a mid-elevation affluent of the right margin of the Olé River (3°18'27.08"N, 8°28'24.36"E) were found on rocks, either in the middle of the stream (the female) or on the shore (the male). The juvenile MNCN 48962 was also found on the moss covering a rock in a stream. Most of the other specimens were found on the upper side of medium-sized leaves, 7–35 cm above the water or ground levels. The adult female MNCN 48956 was perched on a thin branch (1 cm diameter) 1.80 m above the water, while juveniles MNCN 48958 and 48960 were found on the ground. No specimen was observed vocalising or engaging in reproductive behaviours such amplexus, egg deposition, or providing care to offspring.

### Key to adult *Petropedetes* species, modified from Barej et al. (2010)

**Table:** 

1	Toes fully webbed	**2**
–	Toes half or rudimentary webbed	**3**
2	Tympanum distinct, in males ¾ of eye diameter or larger, males with tympanic papillae and dorsal spine on the distal edge of the metacarpal of Finger I, tympanum in females up to ¾ of eye diameter, femoral glands large	***P. perreti***
–	Tympanum small, indistinct in both sexes, males without tympanic papillae, but with dorsal spine on the distal edge of the metacarpal of Finger I, femoral glands large to very large	***P. palmipes***
3	Toes half-webbed	**4**
–	Toes rudimentary-webbed	**5**
4	Tympanum distinct, males with tympanic papillae and dorsal spine on the distal edge of the metacarpal of Finger I, femoral gland line shaped in both sexes	***P. juliawurstnerae***
–	Tympanum indistinct, males without tympanic papillae, dorsal spine on the distal edge of the metacarpal of Finger I absent, femoral gland ovoid	***P. cameronensis***
5	Tympanum small, rounded, in males smaller than eye diameter, tympanic papillae close to upper border of tympanum, femoral glands large	**6**
–	Tympanum of moderate size or large, in males usually large as eye diameter or bigger than the eye	**7**
6	Males with dorsal spine on the distal edge of the metacarpal of Finger I present, keratinised spicules on arms and tympanic borders present, large thenar tubercle, male humeral crest well developed, reaching more than half of the total humeral length, snout slightly pointed	***P. newtonii***
–	Males with metacarpal spines absent, keratinized spicules on arms and tympanic borders absent, relatively small thenar tubercle, humeral crest reaching the half of the total length of the humerus, snout slightly rounded	***P. johnstoni***
7	Femoral gland of moderate size, tympanum in males usually flattened with tympanic papillae closer to the centre than to upper border, dorsal spine on the distal edge of the metacarpal of Finger I present in males	***P. vulpiae***
–	Femoral gland small, tympanic papillae closer to upper border than to the centre, dorsal spine on the distal edge of metacarpal of Finger I present	**8**
8	Femoral gland small, in males 22% of femur length, tympanum size variable from ¾ of the eye diameter to larger than the eye, lowland species	***P. parkeri***
–	Femoral gland very small, in males 16% of femur length, tympanum in males as large as eye diameter, highland species	***P. euskircheni***
